# Surface Modification of Biomedical MgCa_4.5_ and MgCa_4.5_Gd_0.5_ Alloys by Micro-Arc Oxidation

**DOI:** 10.3390/ma14061360

**Published:** 2021-03-11

**Authors:** Piotr Sakiewicz, Krzysztof Piotrowski, Anna Bajorek, Katarzyna Młynarek, Rafał Babilas, Wojciech Simka

**Affiliations:** 1 Department of Engineering Materials and Biomaterials, Silesian University of Technology, Konarskiego 18a, 44-100 Gliwice, Poland; katarzyna.mlynarek@polsl.pl (K.M.); rafal.babilas@polsl.pl (R.B.); 2 Department of Chemical Engineering and Process Design, Silesian University of Technology, M. Strzody 7, 44-100 Gliwice, Poland; krzysztof.piotrowski@polsl.pl; 3 A. Chelkowski Institute of Physics, University of Silesia, Uniwersytecka 4, 40-007 Katowice, Poland; anna.bajorek@us.edu.pl; 4 Department of Inorganic Chemistry, Analytical Chemistry and Electrochemistry, Silesian University of Technology, B. Krzywoustego 6, 44-100 Gliwice, Poland; wojciech.simka@polsl.pl

**Keywords:** MgCaGd alloy, micro-arc oxidation (MAO), corrosion resistance, X-ray photoelectron spectroscopy, SEM, EDS

## Abstract

The aim of this work was to characterize the structure and corrosion properties of the MgCa_4.5_(Gd_0.5_) alloys surface treated by the micro-arc oxidation (MAO) process. The MgCa_4.5_ and MgCa_4.5_Gd_0.5_ alloy samples were processed by MAO in an electrolyte composed of NaOH (10 g/dm^3^), NaF (10 g/dm^3^), NaH_2_PO_4_ (5 g/dm^3^), Na_2_SiO_2_·5H_2_O (10 g/dm^3^) and water. Two different voltages (120 V and 140 V) were used in the MAO process. The alloys protected by an oxide layer formed in the MAO were then the subject of corrosion resistance tests in an environment simulating the human body (Ringer’s solution). After the experiments, the resulting samples were investigated using SEM, XPS and EDS techniques. The addition of Gd affected the fragmentation of the coating structure, thereby increasing the specific surface; higher voltages during the MAO process increased the number and size of surface pores. Corrosion tests showed that the MgCa_4.5_Gd_0.5_ alloys were characterized by low polarization resistances and high corrosion current densities. The studies indicated the disadvantageous influence of gadolinium on the corrosion resistance of MgCa_4.5_ alloys. The immersion tests confirmed lower corrosion resistance of MgCa_4.5_Gd_0.5_ alloys compared to the referenced MgCa_4.5_ ones. The MgCa_4.5_ alloy with the MAO coating established at voltage 140 V demonstrated the best anticorrosion properties.

## 1. Introduction

Magnesium alloys are frequently used in biomedical applications because of their specific properties [1,2,3,4,5,6,7,8,9,10]. However, further improvement is still necessary. For instance, they can be attained by the appropriately oriented modification of the original chemical composition of the alloy. Other methods—used in lieu of or in addition to chemical modification—may involve formation of protective layers on the alloy surface. An external structure (e.g., a hard ceramic layer) may be formed using micro-arc oxidation (MAO) [11,12,13,14,15]. The process is based on the establishment of conditions under which localized micro discharges occur. These result from an electrical disintegration of an oxide layer formed during the process [16]. However, the main challenge is achieving control of the MAO process through the combination and adjustment of appropriate parameter values—striking the required balance between micropores and microfractures/microcracks [17,18]. Nevertheless, development of this external surface, which is advantageous in some applications, can be a technological drawback in others. Other technological concepts are involved, primarily based on the concept of hybrid coating. In this approach, the external surface (with its intrinsic pore and fracture structure resulting from the MAO process) is additionally protected against corrosive agents or electrolytes by a subsequent layer—e.g., physically bound hydroxyapatite, calcium phosphate or a polymer like polylactide working as the sealing agent. It may effectively protect the pore structure against access and penetration, thus make significant elongation of the accepted corrosion level possible. One example is the magnesium alloy AZ91, in which a three-layer coating structure is applied in order to effectively decrease the corrosion rate; MgF_2_ is used for the internal layer, the MAO effect is considered an intermediate layer, and nanostructured hydroxyapatite serves as the external layer. Magnesium alloys with such defined three-layer composite coatings are characterized by higher corrosion resistance, with good adhesion abilities of the natural cells [19,20,21,22]. Other strategies include the addition of some rare earth metals, like gadolinium, into the alloy [23]. Nevertheless, the impact on the living organism (accumulation) is still undergoing medical testing [24].

Voltage influences the quality of the resulting MAO coating. According to the literature, current conditions affect the layer’s thickness and morphology; however, current does not change the layer’s intrinsic structure [25,26]. The coating is thicker and more porous when higher voltages used [26]. In many studies, MAO coatings on Mg-based alloys formed with voltages from within the 200–400 V range (and even up to 500 V) have been analyzed [25,26,27,28,29]. For biomedical applications [30] and for the AZ91D alloy, 120 V and 140 V are frequently used; authors have proven that these current conditions are optimal for corrosion resistance tests [26].

The present work focused on the corrosion behavior of MgCa_4.5_ and MgCa_4.5_Gd_0.5_ alloys in the human body organism’s specific environment—both newly formed alloys and alloys that received some coating of appropriate oxide layers using the micro-arc oxidation technique were studied. The coatings were purposefully designed to decrease the degradation rate of samples in the specific corrosion environment, approaching the conditions provided by blood plasma at 37 °C. These conditions were simulated in vitro by the use of Ringer’s fluid, which is isotonic with respect to human blood.

## 2. Materials and Methods

The research covered two magnesium alloys—MgCa_4.5_ and MgCa_4.5_Gd_0.5_—representing high purity alloys used for medical applications. These demonstrate high biocompatibility, confirmed by the in vitro and in vivo tests [31,32], and can thus be used as implant materials.

The samples used were cylindrical (9 mm diameter; 8.3 mm height) and were appropriately polished with SiC abrasive papers (up to 1000 grit). For these dimensions, the mean total surface of a single sample used for the MAO process was 3.62 cm^2^. Before the MAO process, the raw alloys were rinsed using isopropanol (5 min with ultrasound cleaner), etched with 10% aqueous solution of nitric(V) acid and finally cleaned in demi water.

The MgCa_4.5_ and MgCa_4.5_Gd_0.5_ samples were then subjected to MAO using a high-voltage power supply (KIKUSIU PWR800H, Yokohama, Japan) which was controlled using a PC with Wavy for PWR software (v. 6.0, Yokohama, Japan). The first stage of the study involved the recording of the curve (*U*, *i*) = f (*t*), in an electrolyte composed of NaOH (10 g/dm^3^), NaF (10 g/dm^3^), NaH_2_PO_4_ (5 g/dm^3^), Na_2_SiO_2_·5H_2_O (10 g/dm^3^) and water. Registration was carried out at a current density of 100 mA/cm^2^. Based on test results (not shown), it was found that voltage exceeding 140 V resulted in instability of the MAO process, followed by deterioration of the sample material. It was thus concluded that the main research for experimental identification of optimal MAO process parameters should be done at two different voltages: 120 V and 140 V. The MAO treatment was realized via DC galvanostatic anodization up to the limiting voltage (120 or 140 V) occurrence. After the voltage reached the limit, the treatment was performed under potentiostatic regime. The MgCa_4.5_ and MgCa_4.5_Gd_0.5_ samples, respectively, served as anodes, while a titanium mesh worked as a cathode. In each instance, the process duration was 3 min. During the process, the 200 mL electrolyte solution was constantly mixed with a magnetic stirrer under isothermal conditions at 20 °C. After the anodizing process terminated, the samples were washed in running demi water (18 ΜΩ·cm, Simplicity Water Purification Systems, Millipore SAS, Molsheim, France), air dried and packaged.

For qualitative phase analysis of the alloys, the PANalytical X’Pert PRO diffraction system was used with Cu Kα radiation (λ = 0.15 nm).

Observations of the uncoated alloys’ microstructures were carried out using a Zeiss Axio Observer light microscope at 200× and 500× magnifications.

Both surface morphology and point/region chemical composition of the alloys after micro-arc oxidation (MAO) were analyzed with scanning electron microscope (SEM) using a Supra 35 Carl Zeiss with energy-dispersive X-ray spectroscopy (EDS) EDAX.

Electronic structures of the alloys were determined via X-ray photoelectron spectroscopy (XPS)using a Physical Electronics (PHI 5700/660) spectrometer working in ultra-high vacuum (10^−9^ Torr) conditions with a monochromatic Al Kα X-ray source (1486.6 eV). The surfaces of all specimens were studied after storage under UHV conditions for about 24 h in their as-prepared form, followed by etching with an Ar^+^ beam of 1.5 keV for 30 min. All obtained XPS spectra were calibrated using the C1s peak (BE = 284.8 eV) as the carbon adsorbed on the surface and used as a reference for charge correction. The survey spectra were acquired with a pass energy of 187.85 eV and 0.8 eV/step, whereas all core level lines were measured with a pass energy of 23.50 eV and resolution of 0.1 eV. Deconvolution of core level lines was done via Shirley background and the usual Gauss–Lorentz shape of the lines. All measured spectra were processed with the use of MultiPak 9.4 software in reference to its internal database, and in comparison to the NIST XPS database.

Electrochemical corrosion tests were done in Ringer’s solution at 37 °C using the Autolab 302 N potentiostat controlled by NOVA software. The measurements were done in a three-electrode cell using the Ag/Cl electrode as a reference electrode, a platinum rod as a counter electrode, and a sample as the working electrode. The corrosion resistance was evaluated by recording of the open-circuit potential (*E_OCP_*) variation versus Ag/Cl electrode. The corrosion potential (*E_corr_*), corrosion current density (*j_corr_*) and polarization resistance (*R_p_*) were calculated according to the Stern–Geary method. The values of *β_a_* and *β_c_* (anodic and cathodic slopes, respectively) were determined via Tafel extrapolation (1):

(1) $$j_{corr}=\frac{\beta_{a}\left|\beta_{c}\right|}{2.303\left(\beta_{a}+\left|\beta_{c}\right|\right)}\frac{1}{R_{p}}=\frac{B}{R_{p}}$$

Surface morphology changes within the samples after the electrochemical tests were analyzed using an Axio Observer light microscope (LM) by Zeiss.

Immersion tests were carried out in Ringer’s solution at a stable 37 °C, maintained by a water bath. Observations of morphology changes were carried out at fixed time intervals (1, 2, 3 and 24 h) using the Zeiss Axio Observer LM. Samples were placed in the water bath for an additional 10 min of conditioning time, then connected with heat losses before testing.

## 3. Results

### 3.1. XRD Analysis and LM Observations

**Figure 1:**
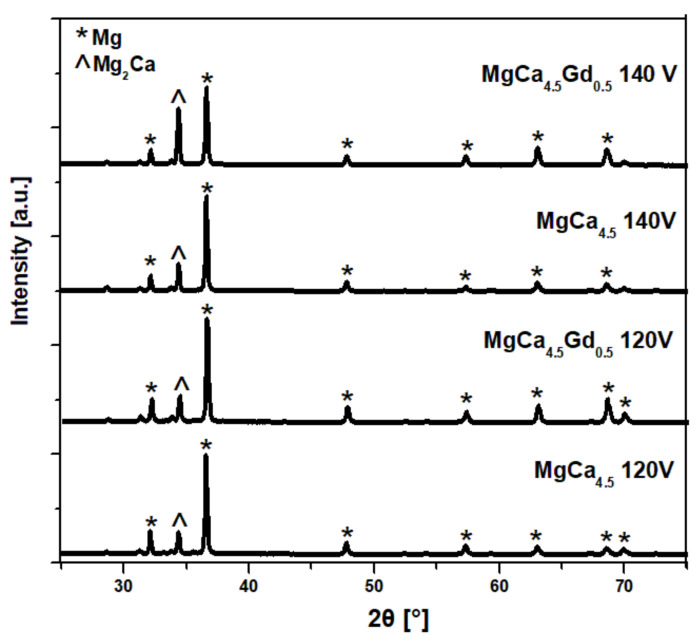
The XRD patterns of MgCa_4.5_Gd_0.5_ and MgCa_4.5_ after micro-arc oxidation (MAO) process done under 140 V and 120 V.

**Figure 2:**
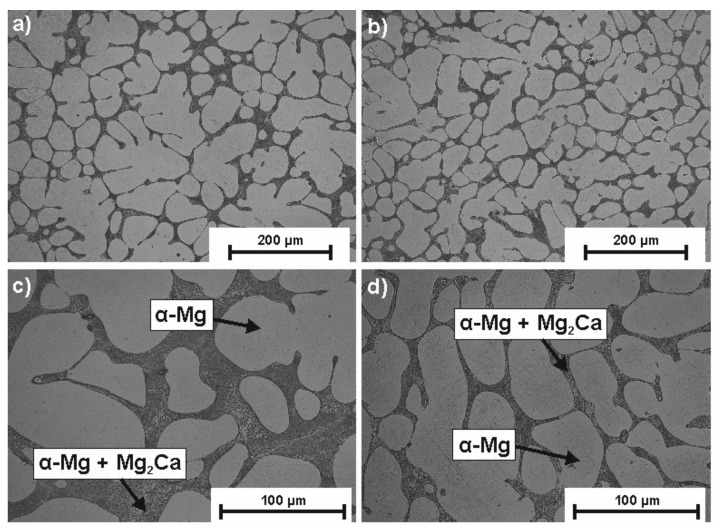
Images of microstructures of (**a**) MgCa_4.5_ (200× magnification), (**b**) MgCa_4.5_Gd_0.5_ (200× magnification), (**c**) MgCa_4.5_ (500× magnification) and (**d**) MgCa_4.5_Gd_0.5_ (500× magnification) alloys in the as-cast state.

Figure 1 shows the XRD patterns of MgCa_4.5_Gd_0.5_ and MgCa_4.5_ alloys after the MAO process done under 140 and 120 V, respectively. It should be noted that two primary phases were identified: α-Mg and Mg_2_Ca. Phase analysis results for the Mg-based alloy with 0.63% of Ca addition were described in the literature, where a pure Mg phase and single Mg_2_Ca peak were also present [33]. In XRD studies, a large angle of incidence of the primary beam was used; therefore, peaks are more intense for the material substrate. As a result, no oxide layer was identified in the analysis [34]. Moreover, application of low voltages (120 V and 140 V) resulted in a very thin oxide layer, which is also confirmed in the literature [26]. Figure 2 shows the microstructures of the uncoated alloys (MgCa_4.5_ (a) and MgCa_4.5_Gd_0.5_ (b)) under 200× magnification. A clear effect of the gadolinium addition on the fragmentation of grain structure was observed. The structures of MgCa_4.5_ and MgCa_4.5_Gd_0.5_ alloys in the as-cast state consisted of a solid solution of α-Mg and eutectics (α-Mg + Mg_2_Ca), located at the grain boundaries and interstices. This is presented in Figure 2c–d using 500× magnification.

### 3.2. SEM Observations and EDS Analysis

**Figure 3:**
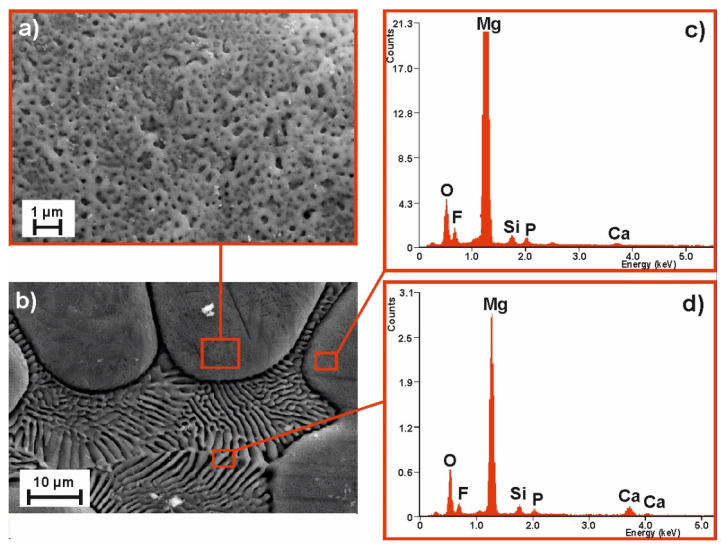
SEM images of the MgCa_4.5_ alloy sample morphology after MAO process under 120 V: (**a**) magnification 500×, (**b**) magnification 5000× and EDS spectra of (**c**) pores, (**d**) grooves.

**Figure 4:**
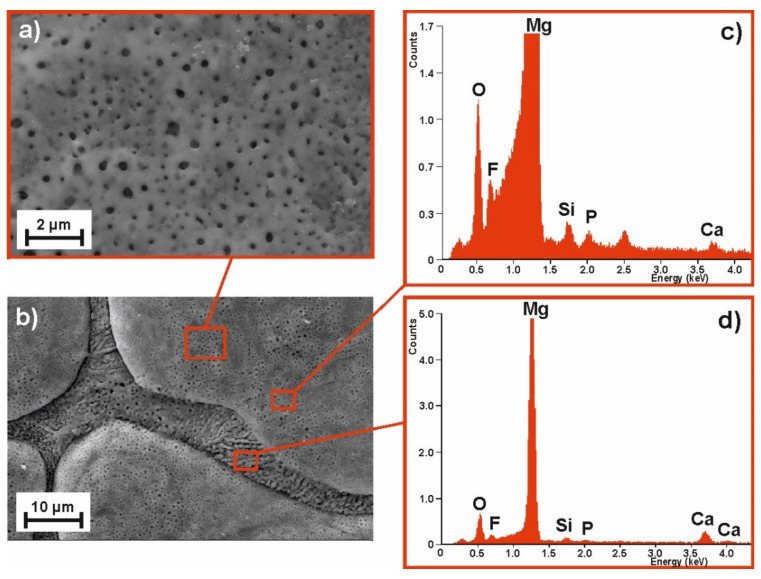
SEM image of the MgCa_4.5_ alloy sample morphology after MAO process under 140 V: (**a**) magnification 500×, (**b**) magnification 5000× and EDS spectra of (**c**) pores, (**d**) grooves.

**Figure 5:**
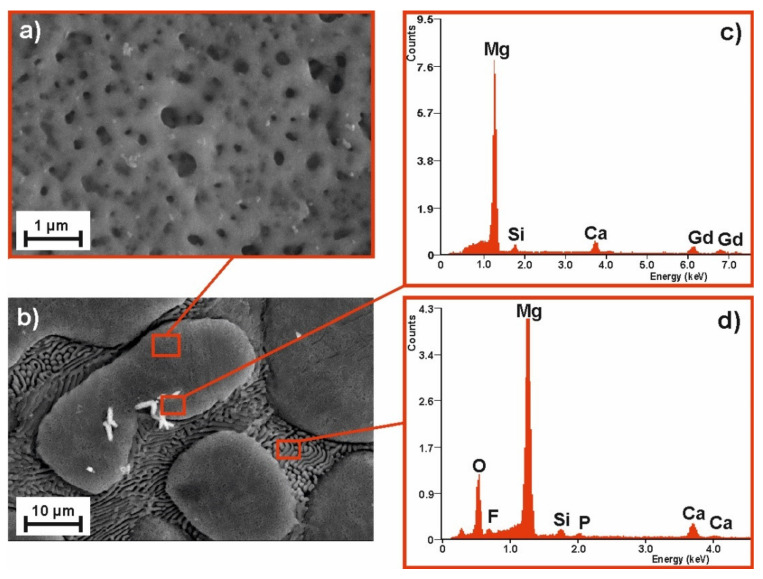
SEM image of the MgCa_4.5_Gd_0.5_ alloy sample morphology after MAO process under 120 V: (**a**) magnification 500×, (**b**) magnification 5000× and EDS spectra of (**c**) pores, (**d**) grooves.

**Figure 6:**
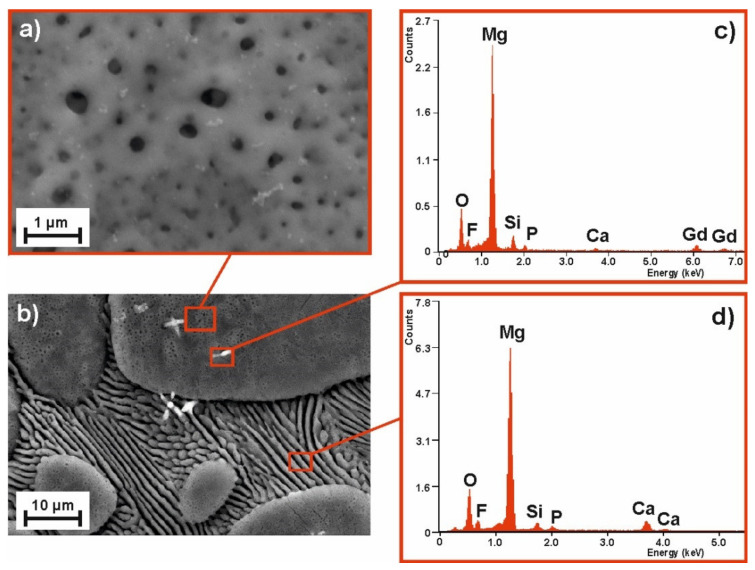
SEM image of the MgCa_4.5_Gd_0.5_ alloy sample morphology after MAO process under 140 V: (**a**) magnification 500×, (**b**) magnification 5000× and EDS spectra of (**c**) pores, (**d**) grooves.

To compare the samples’ surface quality after the MAO process for two alloy compositions and the two voltages applied, the SEM images of their surface morphology (with the corresponding EDS spectra) are compiled as follows: Figure 3—MgCa_4.5_ (120 V); Figure 4—MgCa_4.5_ (140 V); Figure 5—MgCa_4.5_Gd_0.5_ (120 V); and Figure 6—MgCa_4.5_Gd_0.5_ (140 V). The surface morphologies of all analyzed coatings were porous, as is demonstrated in detail in Figure 3a, Figure 4a, Figure 5a and Figure 6a (2500× magnification). According to the literature, MAO coatings on magnesium alloys demonstrate porous structures [22]. This is a typical result of oxide coatings on Mg-based alloys. This is, however, advisable for specific biomedical applications because of the improvement in osseointegration of the developed surface area—particularly the way in which pores support bioconnection with bone tissue. Figure 3a, Figure 4a, Figure 5a and Figure 6a show detailed versions of areas marked in Figure 3c, Figure 4c, Figure 5c and Figure 6c, where a 500× magnification was applied. Morphologies of the materials studied (despite the MAO coatings applied) were also characterized by a visible phase separation of the solid solution of α-Mg and eutectics (α-Mg + Mg_2_Ca). Lack of homogeneity resulted from the low voltages applied, which only produced very thin coatings [26]. The effect of higher process voltages on pore sizes can be clearly observed in Figure 3a, Figure 4a, Figure 5a and Figure 6a, which show a larger number of pores within the structure of alloys formed under 140 V. The influence of higher voltage on pore diameter increase on the Mg-Ca substrate was also described in the literature. For example, pore diameter was 0.4 µm at 300 V, 1.2 µm at 360 V and 1.5 µm at 400 V [35]. In another work, the difference in pore size observed within MAO coatings formed on the Mg-Zn-Ca substrate with voltages of 120 V and 140 V was indicated [30].

The EDS results related to the areas marked in Figure 3c,d, Figure 4c,d, Figure 5c,d and Figure 6c,d (magnification 5000×) are presented for the coatings formed on: b) grain and d) grain boundary, respectively. Both areas of the MAO coating on the MgCa_4.5_ substrate alloy have nearly the same chemical composition with respect to the following elements: Mg, Ca (derived from the alloy substrate), O (from the oxide layer formed), Si, F and P (from the working electrolyte solutions used during the micro-arc oxidation process) (Figure 3c,d, Figure 4c,d). Slight differences in the EDS spectra can be observed in the case of MgCa_4.5_Gd_0.5_ alloys, where there were identified: Mg, Ca, Gd (from the alloy substrate). Gd was especially observable in porous areas (Figure 5c, Figure 6c). Oxygen content from the oxide layer (and Si, F and P from the electrolyte solutions) was also identified, as in the compositionally simpler MgCa_4.5_ alloys.

### 3.3. XPS Analysis

**Figure 7:**
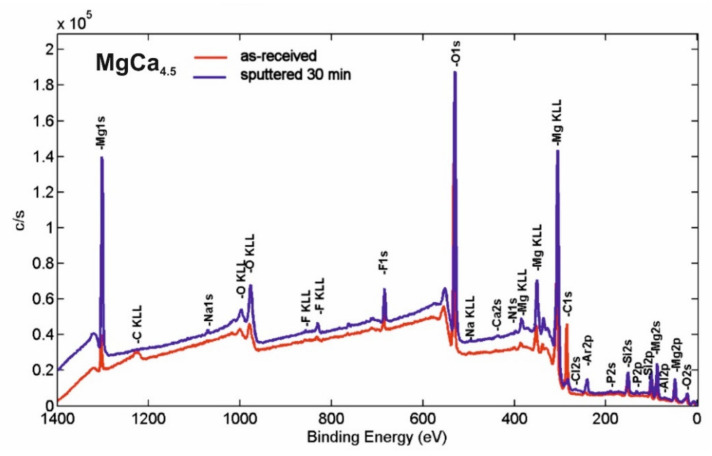
XPS survey spectra of the MgCa_4.5_ alloy sample after micro-arc oxidation (140 V) in as-received state and after etching.

**Figure 8:**
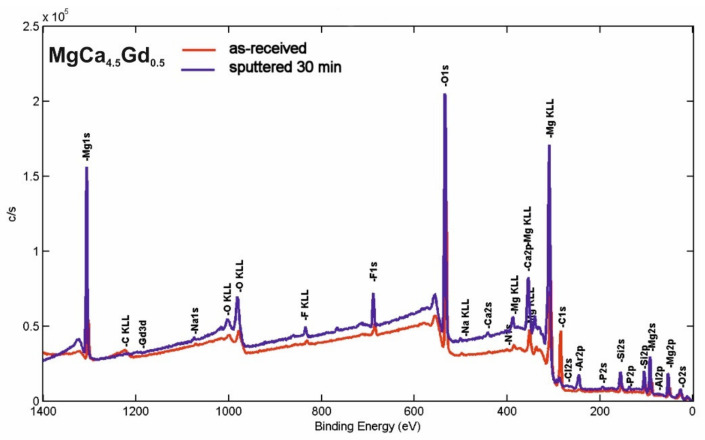
XPS survey spectra of the MgCa_4.5_Gd_0.5_ alloy sample after micro-arc oxidation (140 V) in as-received state and after etching.

The XPS survey spectra of the surface products after micro-arc oxidation under 140 V for MgCa_4.5_ and MgCa_4.5_Gd_0.5_ alloys are presented in Figure 7 and Figure 8, respectively. The quantitative characteristics of the samples in respect to the individual elements determined from these curves are listed in Table 1. The surface elements identified instrumentally after micro-arc oxidation were: Mg, Ca and Gd as the original alloy substrates; O, P, Cl, Na, F and Si as the products derived from the electrolyte and MAO procedures; Al, Si and C as the impurities introduced during sample preparation; and Ar from the etching process. All of this corresponded with the method of alloy preparation and was consistent with XPS results reported in the literature [36,37]. Based on the obtained results for both MgCa_4.5_ and MgCa_4.5_Gd_0.5_ alloys, an increase in the oxygen content was observed, especially due to the oxide layers formed. In addition, the presented results indicated the appearance of fluorine after the MAO process. Moreover, it may be assumed that oxygen difluoride or magnesium fluoride were formed after the oxidation process. Similarly, in the studies of Gan et al. [37], after the coating process on magnesium alloy (with Ca and P), researchers noted the formation of a layer consisting of MgF_2_, MgO, Mg_3_(PO_4_)_2_, Ca_2_P_2_O_7_ and CaHPO_4_. The presence of fluoride was also confirmed for all samples by EDS analyses. One work indicated F content as an effect of the MAO coating process [38].

In both alloys (MgCa_4.5_ and MgCa_4.5_Gd_0.5_), reduction of the carbon content after the ion cleaning was not surprising; it was the result of removing surface impurities with Ar^+^ beams.

**Figure 9:**
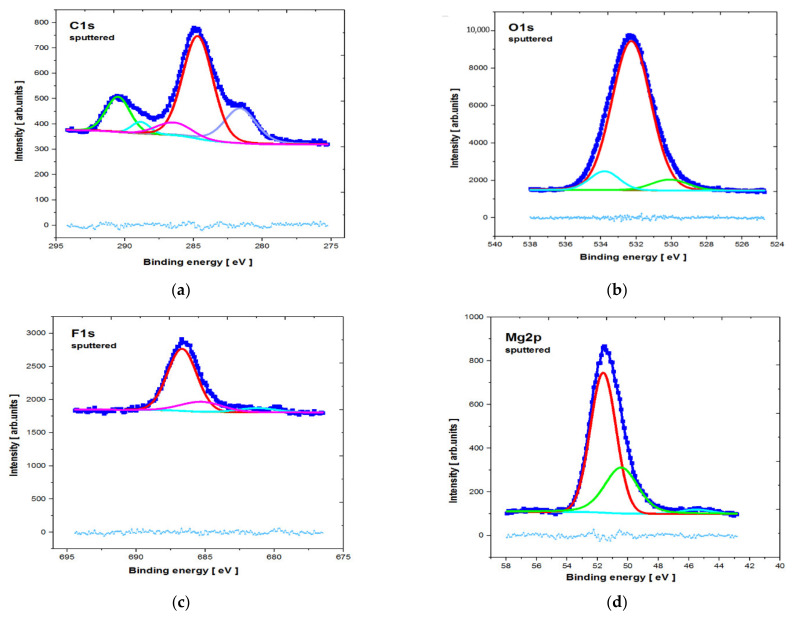
XPS core level lines of MgCa_4.5_ alloy directly after the micro-arc oxidation and after etching (**a**) C1s; (**b**) O1s; (**c**) F1s; (**d**) Mg2p.

**Figure 10:**
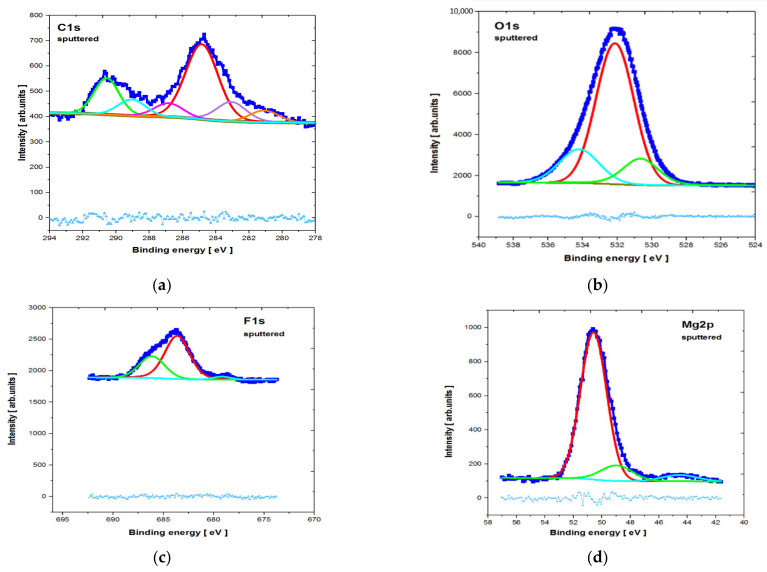
XPS core level lines of MgCa_4.5_Gd_0.5_ alloy directly after micro-arc oxidation and etching (**a**) C1s; (**b**) O1s; (**c**) F1s; (**d**) Mg2p.

The core level lines acquired after ion bombardment are shown in Figure 9 and Figure 10. The quantitative breakdown interphase distribution of the narrow scan spectra of the elements is presented in Table 2 and Table 3, respectively. The slight energy shift into higher binding energy of all of the core level lines (compared to the database) resulted from surface modification by the argon beam.

The C1s line for both analyzed samples was quite complex after etching. The line clearly observable in the middle of the spectra (with the highest intensity) probably represents C—the surface component derived from the environment (BE ≈ 284.8 ± 0.3eV). This is similar to some results described in the literature [37]. Other photoemission lines in the lower BE range may be attributed to various carbides, whereas those in higher BE range may be attributed to various carbonates, including CaCO_3_ (BE ≈ 289.9 ± 0.3eV).

The O1s core level lines can mostly be assigned to MgO states (BE ≈ 532.1 ± 0.3eV) [37]. This is also indicated in the literature, where the presence of the MgO phase was identified after the MAO process for MgCa alloys [35]. The presence of phosphorus(V) oxide (e.g., P_2_O_5_—BE ≈ 534.2 ± 0.3eV) and calcium oxide (e.g., CaO (BE ≈ 529.2 ± 0.3eV)) may also be noted, but the latter peak overlapped with the lattice oxygen. For the MgCa_4.5_Gd_0.5_ sample, the oxygen line may have partly overlapped with Gd_2_O_3_ and, therefore, the percentage contribution of the low BE line increased (by 13.44%) compared to the MgCa alloy (by 8.56%). The presence of Gd_2_O_3_ is also indicated by the XPS results of the formation of the gadolinium oxide layer on the magnesium alloy substrate [39]. The influence of the adsorbed Mg(OH)_2_ on O1s line in both studied specimens (especially in higher BE) may also be relevant. This was also described in the literature [40].

The F1s line was dominated by the MgF_2_ states (BE ≈ 686.7 ± 1 eV) [37], overlapped with CaF_2_ (BE ≈ 685.3 ± 1 eV) states. NaF contribution after the MAO process must also be considered, but—as was evidenced by the quantitative analysis—the Na1s states were barely observed This means that, compared to calcium and magnesium fluorides, the NaF states were rather slight. The Mg2p line exhibited significant extent of the MgF_2_ contribution; the most intense photoemission peaked with BE ≈ 51.6±1 eV, which was, however, partially overlapped with MgO (BE ≈ 50.4 ± 1 eV) states. Similar results for the presence of MgF_2_ and MgO were described for pure magnesium with an MAO coating by Gan et al. [37]. Additionally, in another article, XPS results indicated the presence of MgF_2_ and MgO for the formed gadolinium oxide coating on pure magnesium [39].

### 3.4. Electrochemical Measurements

**Figure 11:**
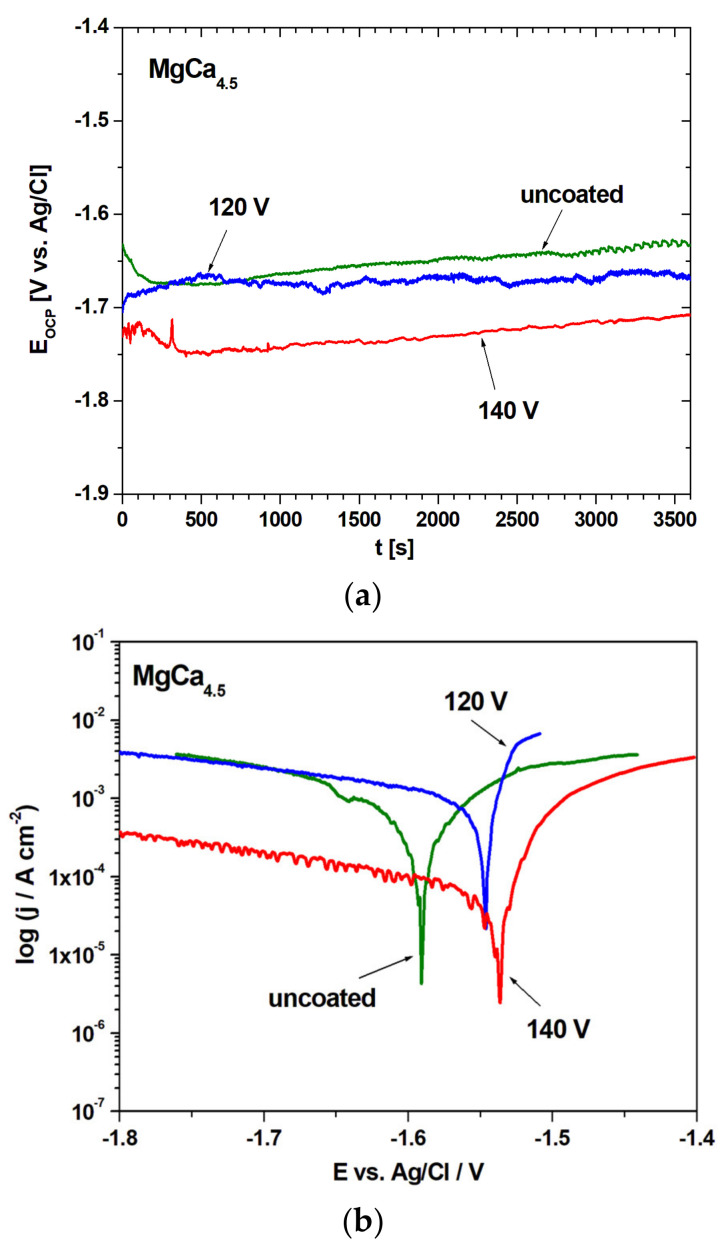

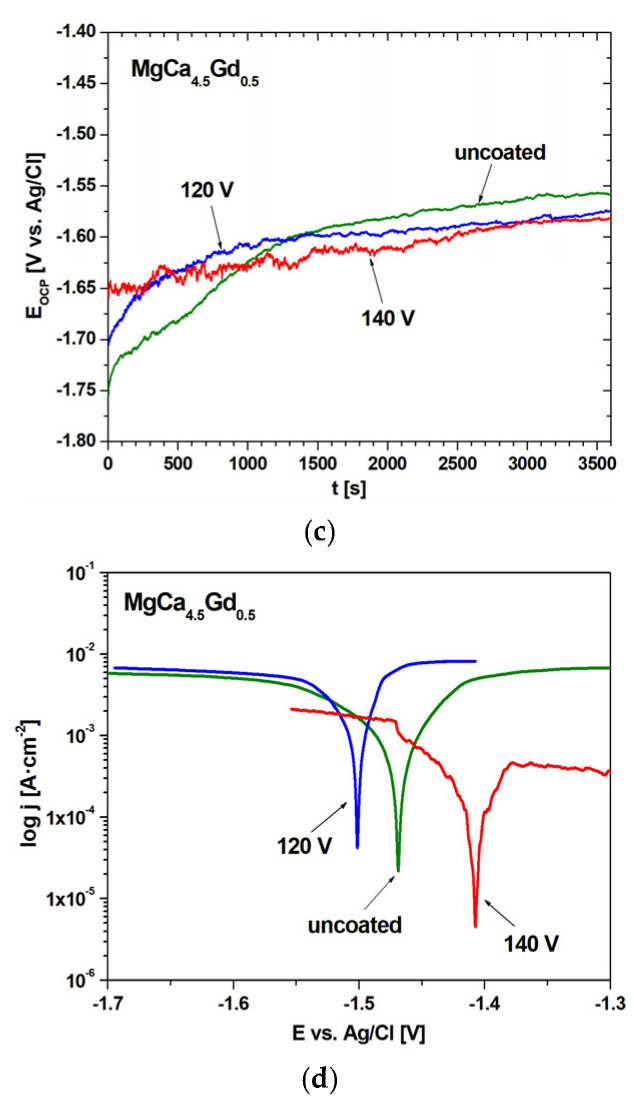
Open-circuit potential changes with time (**a**,**c**) and polarization curves (**b**,**d**) in Ringer’s solution (37 °C, MgCa_4.5_ and MgCa_4.5_Gd_0.5_ alloys).

The electrochemical tests involving the stationary open-circuit potential change as a function of time and polarization curves are presented in Figure 11 for MgCa_4.5_ (a,b) and for MgCa_4.5_Gd_0.5_ (c,d), for both voltages (120 V and 140 V) and uncoated alloys. The values of open circuit potential (E_OCP_) for uncoated MgCa_4.5_ and MgCa_4.5_Gd_0.5_ alloys were the most shifted toward the positive values. However, the results of the stationary open-circuit potential for MgCa_4.5_Gd_0.5_ were similar for all samples. In the case of polarization curves, the alloys coated via MAO at 140 V were shifted toward lower corrosion current density and better corrosion potential values than alloys coated via MAO at 120 V or uncoated materials.

The results of open-circuit potential (E_OCP_), corrosion potential (E_corr_), anodic and cathodic Tafel slopes (β_a_, β_c_), polarization resistance (R_p_) and corrosion current density (j_corr_) are presented in Table 4.

Generally, more favorable open circuit potential was observed for the alloys with added gadolinium. The most positive value of E_corr_ potential was achieved (−1407 mV) for MgCa_4.5_Gd_0.5_—140 V, while the most negative value (−1591 mV) corresponded to the MgCa_4.5_—uncoated case. The highest polarization resistance (194.87 Ω∙cm^2^) was recorded for MgCa_4.5_—140 V, whereas the lowest (6.94 Ω∙cm^2^) was recorded for MgCa_4.5_Gd_0.5_—120 V. The R_p_ values were more favorable for MgCa_4.5_ compositions. Similarly, the alloys with the MAO coating filled under 140 V turned out to be better than those filled under 120 V conditions. The corrosion current density was the lowest (0.04 mA/cm^2^) for MgCa_4.5_—140 V, and the highest (2.08 mA/cm^2^) for MgCa_4.5_Gd_0.5_—uncoated. In case of j_corr_, more favorable values were identified for MgCa_4.5_ alloys.

**Figure 12:**
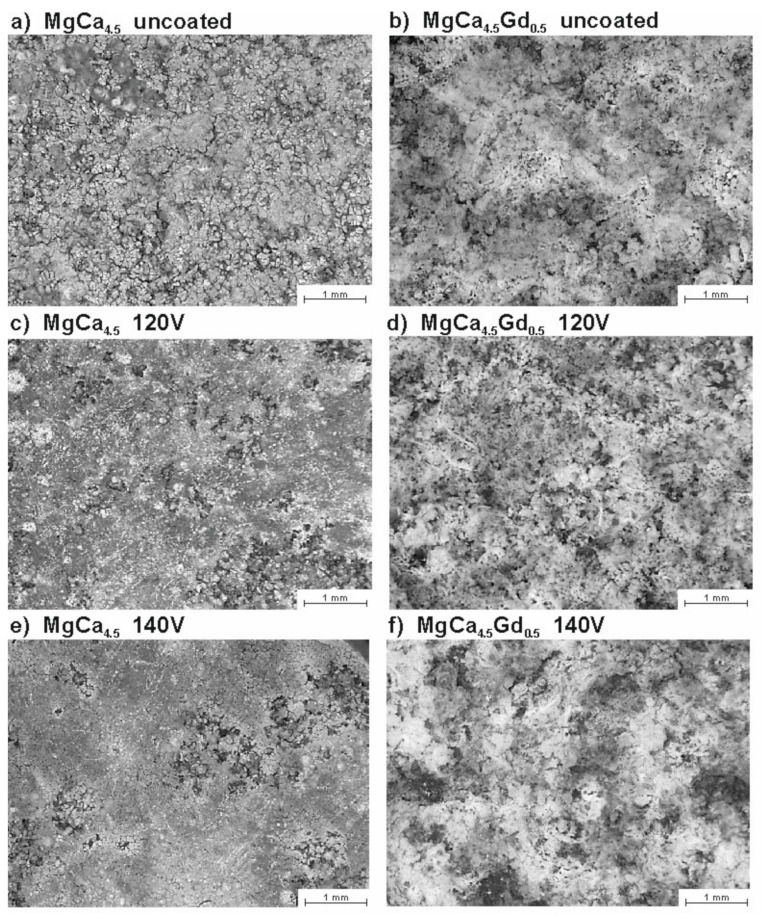
Surface morphology of the MgCa_4.5_ (**a**,**c**,**e**) and MgCa_4.5_Gd_0.5_ (**b**,**d**,**f**) alloys after electrochemical tests in Ringer’s solution.

The surface morphologies of the studied alloys after electrochemical tests are presented in Figure 12. In Figure 12a,c,e—corresponding to MgCa_4.5_ alloys, contrary to MgCa_4.5_Gd_0.5_ alloys—the metallic gloss and fragmentation of the structure can be observed. The presented morphology images are consistent the results presented in the literature [41] concerning corrosion tests for MgCa_0_._7_ and MgCa_0_._9_ alloys. The authors demonstrated that the formed layer of corrosion products cracked, which can also be observed in this work (especially for the MgCa_4.5_ uncoated alloy). Moreover, Mareci et al. [42] described the formation of significantly surface-cumulated corrosion products for Mg_0_._6_Ca and Mg_0_._9_Ca alloys. The surfaces of MgCa_4.5_Gd_0.5_ samples were covered by relatively thick layers of some corrosion products (Figure 12b,d,f). The literature [43] confirmed the formation of corrosion products in the form of magnesium oxides and other compounds (including gadolinium) for Mg_0.5_Ca_x_Gd alloys (x = 0, 0.5, 1, 1.5, 2, 3). The authors noted that the compounds formed on the surface were unstable and cracked [43].

In conclusion, the general improvements in E_OCP_ and E_corr_—as a result of the addition of gadolinium—and polarization resistance of MAO under 140 V can be observed, which is consistent with literature data indicating some improvement in corrosion resistance after such modifications [30,32,44]. The advantageous effect of the Gd addition in the electrochemical tests results was also verified for the Mg-Zn-Y alloys (as presented by Zhang et al. [45]) and MgCa alloys (as reported by Fernandes et al. [46]). Moreover, the influence of Gd addition into magnesium alloys resulting in improved corrosion resistance was described by Chen et al. [47]. Additionally, according to [39], the corrosion susceptibility of alloys with lower Gd content probably resulted from a larger volume of the CaMg_2_ phase. Moreover, on the uncoated Mg-Ca-Gd alloys, the passive layer formed is more stable (compared to the MgCa alloys alone). The discrepancy between the favorable E_OCP_ and E_corr_ values for the alloys with the MAO coatings produced under 120 V and high polarization resistance with low corrosion current density for the applied voltage of 140 V is probably related to the presence of the obtained coatings. Narayanan et al. [22] indicated that the main technical limitation observed during micro-arc oxidation coating under high voltages is the formation of corrosive media inside the pores. On the other hand, other literature data described similar (more porous and open) structures as more advantageous for the free flow of electrolyte, supplementing the oxygen during passivation [48]. It should also be noted that the discrepancy between potentials and corrosion current densities suggests better corrosion resistance for the MgCa_4.5_ alloys. This is because the E_OCP_ and E_corr_ are thermodynamic parameters, whereas j_corr_ is a kinetic one.

**Figure 13:**
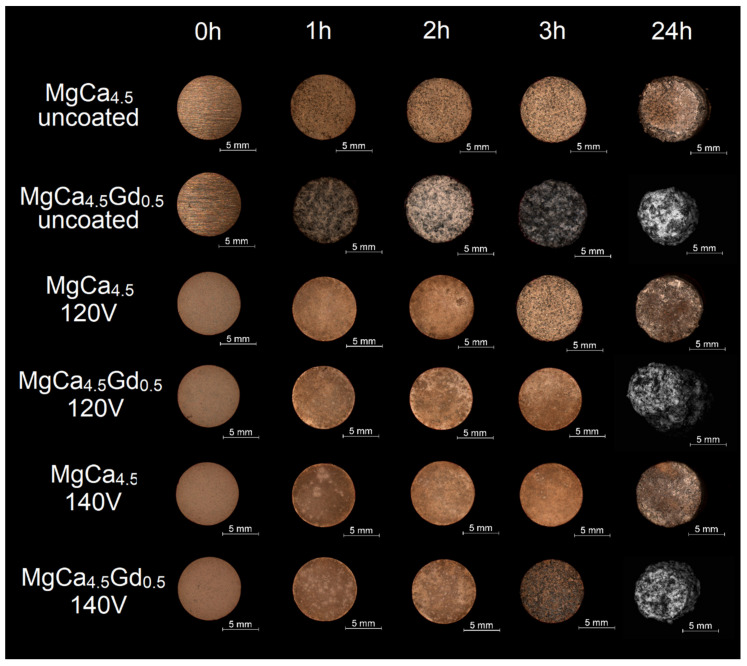
Optical micrographs of the MgCa_4.5_ and MgCa_4.5_Gd_0.5_ alloys after immersion tests in Ringer’s solution at 37 °C.

To verify the obtained electrochemical corrosion resistance measurements, immersion tests were carried out. The resulting changes in the morphologies of studied alloys for individual time intervals are demonstrated in Figure 13. Based on the images collected by the light microscope, it was observed that the uncoated MgCa_4.5_Gd_0.5_ alloy was the least resistant. For this composition, the surface changes appeared after 1 h of the test. The alloys with the gadolinium addition and MAO coatings were characterized by better corrosion resistance compared to the same alloy without the coating. However, after 24 h the uncontrolled degradation effects could be observed—especially as a visible change in the cross-sectional geometry of the sample. In terms of corrosion resistance, the MgCa_4.5_ alloy with the coating formed under 140 V proved to be the best; daily tests showed only slight changes in the cross-section area (compared to the alloys with Gd addition). After 24 h, the uncoated MgCa_4.5_ alloy was beginning the resorption process. The alloy with the coating formed under 120 V demonstrated no clear signs of the geometry variation.

Based on the results of immersion tests and the literature reports, it can be concluded that—after the first hour—results were comparable to electrochemical tests, where the formation of corrosion products on the sample surface was observed [44]. There are literature data concerning immersion tests with detailed characteristics of surface topography of various MgCa(Gd) alloys [42,44,46]. In one report [42], where the same immersion test parameters were applied, extensive damage to the test surface with nonuniform corrosion attack was described for Mg_0_._6_Ca and Mg_0_._9_Ca. This is consistent with the authors’ own results, where the cross-sections of tested samples were not regular after 24 h of the test. The corrosion mechanism was described in the literature [49], where the electrolyte reacted with the substrate through the pores in the magnesium-based coatings, which led to delamination of the coating. In cases in which massive and unstable corrosion products form on the alloys (with the addition of gadolinium), this could occur. The literature provides data on the improvement of corrosion resistance of alloys with MAO coatings established on the Mg-based alloys. These data are also confirmed in this work [50,51].

## 4. Conclusions

The oxide layers formed on both alloys after the MAO process consisted mainly of magnesium, representing the main phase within all samples studied. According to the XPS and EDS spectra, the interphase distribution of the elements from the electrolyte environment in the coating process formed highly biocompatible compounds (e.g., MgF_2_). The MAO coatings applied on the grains α-Mg and eutectics (α-Mg + Mg_2_Ca) demonstrated identical qualitative chemical composition, regardless of the current electroconditions applied. The voltages (120 V and 140 V) were relatively low; thus, the layer formed was rather thin and separation between the phases of the substrate was visible. The influence of gadolinium on the fragmentation development observed within the outer layer structure of the samples was demonstrated. The effect of increased voltage during the MAO process on the quantity and size of pores formed within the outer layer was also indicated. Highly-developed specific surface areas—and some knowledge about their porosity—allow for the rational design of alloys characterized by high, modifiable osseointegration properties. Furthermore, the addition of gadolinium to the MgCa_4.5_ alloy influenced the shift of the open-circuit potential and corrosion potential to positive values during the electrochemical tests. However, the MgCa_4.5_Gd_0.5_ alloys were characterized by low polarization resistances and high corrosion current densities. The immersion tests confirmed lower corrosion resistance of MgCa_4.5_Gd_0.5_ alloys compared to MgCa_4.5_ alloys. The use of MAO coatings significantly contributed to the improvement of corrosion resistance; however, in cases of gadolinium addition, the degradation and geometrical changes were visible after 24 h of immersion tests. The MgCa_4.5_ alloy with the MAO coating formed under 140 V was characterized by the best anticorrosion properties. This makes it possible to clearly state that the modification of these alloys’ surfaces (with the use of appropriate combinations of MAO parameter values) contributes to the indirect control of resorption processes for bioresorbable alloys.

## Figures and Tables

**Table 1:** Quantitative interphase distribution of elements identified on the studied MgCa_4.5_ and MgCa_4.5_Gd_0.5_ alloy surfaces in as-received and etched states.

**MgCa_4.5_**													
	C1s	O1s	N1s	F1s	Na1s	Mg2s	Ca2s	Al2p	Si2p	P2p	Cl2p	Ar2p	
As-received	35.73	42.31	0.85	2.37	0.12	10.85	0.33	0.44	6.75	0.17	0.09	-	
Etched	8.03	54.35	0.22	4.43	0.50	17.88	1.37	0.45	9.49	0.76	0.09	2.42	
**MgCa_4.5_Gd_0.5_**													
	C1s	O1s	N1s	F1s	Na1s	Mg2s	Ca2s	Al2p	Si2p	P2s	Cl2p	Ar2p	Gd3d
As-received	36.69	40.83	0.74	2.69	0.32	11.06	0.72	0.31	6.24	0.31	0.05	-	0.03
Etched	6.46	53.48	0.04	4.68	0.50	20.20	1.20	0.26	9.30	0.90	0.00	2.80	0.18

**Table 2:** Quantitative breakdown interphase distribution of the narrow scan spectra of the elements of the studied etched surface for MgCa_4.5_.

Line	Components						
	Position [eV]	Separation [eV]	FWHM	Height	%Gauss	%Area	χ-sqr
O1s (etched)	530.10	0	2.45	582	99	5.86	0.97
	532.27	2.18	2.58	7973	95	86.09	
	533.79	3.70	1.96	997	99	8.04	
C1s (etched)	281.61	0	2.76	138	70	21.12	2.45
	284.69	3.08	2.58	406	90	53.51	
	286.43	4.82	3.00	50	100	7.36	
	288.85	7.24	1.54	46	100	3.46	
	290.50	8.89	2.04	139	90	14.55	
F1s (etched)	681.23	0	3.19	62	90	6.78	1.37
	685.27	4.04	3.52	150	100	17.20	
	686.68	5.44	2.48	937	100	76.02	
Mg2p (etched)	45.51	0	2.37	20	100	2.40	1.59
	50.44	4.93	2.58	210	73	31.59	
	51.62	6.12	1.99	641	100	66.01	

**Table 3:** Quantitative breakdown interphase distribution of the narrow scan spectra of the elements of the studied etched surface for MgCa_4.5_Gd_0.5_.

Line	Components						
	Position [eV]	Separation [eV]	FWHM	Height	%Gauss	%Area	χ-sqr
O1s (etched)	530.65	0	2.41	1293	76	13.44	2.03
	532.13	1.48	2.58	6878	100	68.52	
	534.20	3.55	2.70	1649	90	18.04	
C1s (etched)	281.06	0	2.13	48	100	6.6	1.87
	283.07	2.00	2.09	78	80	11.60	
	284.85	3.79	2.31	300	90	47.11	
	286.84	5.78	2.04	57	90	7.92	
	289.06	8.00	2.13	65	100	8.96	
	290.57	9.51	1.87	140	90	17.80	
F1s (etched)	679.19	0	2.14	47	100	3.00	1.81
	683.63	4.44	2.92	684	80	64.99	
	686.14	6.95	2.96	348	90	32.01	
Mg2p (etched)	44.55	0	2.87	37	100	4.79	1.56
	48.98	4.44	2.62	91	100	10.75	
	50.59	6.04	2.16	865	100	84.46	

**Table 4:** The polarization tests of MgCa_4.5_ and MgCa_4.5_Gd_0.5_ alloys in Ringer’s solution at 37 °C (E_OCP_: open-circuit potential; E_corr_: corrosion potential; β_a_, β_c_: anodic and cathodic Tafel’s slopes; R_p_: polarization resistance; j_corr_: corrosion current density).

Voltage [V]	E_OCP_ [mV]	E_corr_ [mV]	|β_a_| [mV/dec]	|β_c_| [mV/dec]	R_p_ [Ω∙cm^2^]	j_corr_ [mA/cm^2^]
			MgCa_4.5_			
uncoated	−1633	−1591	141	108	40.25	0.66
120	−1666	−1546	45	15	13.78	0.35
140	−1708	−1536	207	21	194.87	0.04
			MgCa_4.5_Gd_0.5_			
uncoated	−1558	−1468	252	121	17.07	2.08
120	−1574	−1501	62	33	6.94	1.34
140	−1583	−1407	110	104	70.24	0.33

## Data Availability

Data sharing is not applicable to this article.
